# Clinical significance of frusemide stress test in predicting the severity of acute kidney injury

**DOI:** 10.1590/2175-8239-JBN-2021-0003

**Published:** 2021-04-19

**Authors:** Arun Gokul Pon, Raveendran Vairakkani, Edwin Fernando Mervin, Nagalakshmi Dhanapal Srinivasaprasad, Thirumalvalavan Kaliaperumal

**Affiliations:** 1 Government Stanley Medical College and Hospital, General Medicine, Chennai, Tamilnadu, India.; 2 Government Stanley Medical College and Hospital, Nephrology, Chennai, Tamilnadu, India.

**Keywords:** Acute Kidney Injury, Furosemide, Exercise Test, Lesão Renal Aguda, Furosemida, Teste de Esforço

## Abstract

**Introduction:**

The outcomes of Acute Kidney Injury (AKI) remain dismal even today, owing in part due to the lack of an ideal biomarker for detecting renal damage early enough. We conducted this pilot study to determine the clinical significance of Frusemide Stress Test (FST) to predict the severity of AKI.

**Methods:**

A total of 80 patients with AKI-KDIGO (Kidney Disease: Improving Global Outcomes) stage 1 or stage 2 underwent FST by administering a bolus dose of frusemide (1mg/kg for frusemide naïve and 1.5mg/kg for prior frusemide exposure in the past week), and urine output was then measured for the next two hours with volume replacement as desirable. The progression to AKI-KDIGO stage 3 within 14 days of FST was studied as the primary outcome. The composite end point of achieving AKI-KDIGO stage 3 or death within 14 days of FST was studied as the secondary outcome.

**Results:**

Out of 80 patients, 28(35%) patients met the primary outcome, and 34(42.5%) patients met the secondary composite outcome. Except for baseline Chronic Kidney Disease (CKD) status (p=0.018), other demographic characteristics were comparable between progressors and non-progressors group. Using receiver operating characteristics (ROC) curve analysis, a cumulative 2-hour post-FST urine output of ≤300 mL predicted progression to stage 3 AKI with 82.14% sensitivity, 82.69% specificity, and AUC of 0.89±0.03 (p<0.0001).

**Conclusion:**

The FST showed promising results as a novel tubular biomarker to identify progression to severe AKI with good predictive ability.

## Resumo

**Introdução:**

Os desfechos da Lesão Renal Aguda (LRA) permanecem desanimadores ainda hoje, em parte pela falta de um biomarcador ideal para detectar danos renais com a devida antecedência. Realizamos este estudo piloto para determinar a importância clínica do Teste de Estresse com Furosemida (TEF) em prever a gravidade da LRA.

**Métodos:**

Um total de 80 pacientes com LRA-KDIGO estágio 1 ou 2 foram submetidos ao TEF pela administração de uma dose em bolus de furosemida (1mg/kg para pacientes virgens de furosemida e 1,5mg/kg para exposição prévia à furosemida na semana anterior). O débito urinário foi então medido durante as duas horas seguintes com reposição de volume conforme desejável. A progressão para LRA-KDIGO estágio 3 dentro de 14 dias de TEF foi estudada como principal desfecho. O desfecho composto de atingir a LRA-KDIGO estágio 3 ou óbito em 14 dias após TEF foi estudado como desfecho secundário.

**Resultados:**

Dos 80 pacientes, 28 (35%) atingiram desfecho primário, e 34 (42,5%) pacientes atingiram o desfecho composto secundário. Exceto pelo estado basal da Doença Renal Crônica (DRC) (*p*=0,018), outras características demográficas foram comparáveis entre o grupo progressores e não progressores. Usando a análise da Curva Característica de Operação do Receptor (ROC), um débito urinário cumulativo de 2 horas pós-TEF de ≤300 mL previu a progressão para estágio 3 da LRA com 82,14% de sensibilidade, 82,69% de especificidade, e AUC de 0,89±0,03 (p<0,0001).

**Conclusão:**

O TEF mostrou resultados promissores como novo biomarcador tubular para identificar progressão para LRA grave com boa capacidade preditiva.

## Introduction

Despite making huge strides in the science of nephrology, the morbidity and mortality burden of acute kidney injury (AKI) - the most common kidney affliction - remains substantial even today^1^^,^ 2. Apart from addressing the underlying etiology, optimizing renal perfusion, maintaining volume status, discontinuing or avoiding nephrotoxins, proactive drug dosage adjustment, and if indicated renal replacement therapy (RRT) form the crux of AKI management in almost all, effective therapeutics are lacking in most circumstances^1^^,^ 2. One of the reasons cited for this conundrum being the contemporary diagnosis of AKI using creatinine and urine output criteria, gets established only after significant damage to the renal parenchyma has occurred^2^^-^ 4. To improve patient outcomes with AKI, interventions need to be made during the "window of opportunity", which includes the time frame during which kidney injury exists without marked change in creatinine due to the presence of renal reserve^3^. To recognize this early AKI, tedious research has been done over the past decade to identify an ideal biomarker^3^^,^ 5 ^,^ 6. These structural damage biomarkers studied so far have shown variable ability in identifying AKI early, aside from their problems of cost, bedside availability, and methodological difficulties^2^^,^ 6.

Recently, there has been an interest in testing functional biomarkers to risk-stratify early AKI patients. To assess integrated tubular function, the pharmacokinetic properties of frusemide appears promising^4^^,^ 5. Frusemide has been used informally for years by physicians to administer diuretic challenge in oliguric patients and failure to respond was considered ominous. The standardized version of this bedside practice by utilizing the diuretic response to a standard weight-based dose of frusemide to predict AKI progression constitutes the Frusemide Stress Test (FST) as demonstrated in the seminal paper by Chawla LS *et* al^4^^,^ 5. An increase in urine output in response to diuretic challenge is believed to reflect tubular integrity and hence less tubular injury^5^.

In our study, we analyzed the role of FST in predicting AKI severity in our population.

## Methods

This study was a prospective, open labelled pilot study conducted at the Government Stanley medical college and hospital, Chennai, India, between April 2018 to September 2018 after approval by the Institutional Ethics Committee of the hospital, dated 13-04-2018.

### Patient Selection

Patients above 18 years of age admitted to intensive care unit with either AKI-KDIGO stage 1 (an increase in creatinine ≥0.3 mg/dL within past 48 hours or an increase from baseline value by 1.5-1.9 times or a urine output < 0.5 mL/kg/hr for 6-12 hours) or AKI-KDIGO stage 2 (an increase in creatinine from baseline value by 2.0 -2.9 times or a urine output < 0.5 mL/kg/hr for ≥12 hours) who were assessed to be euvolemic or hypervolemic in the opinion of the treating team and had an indwelling bladder catheter were eligible for the study. Patients with baseline advanced chronic kidney disease (CKD) defined by eGFR < 30 mL/min/1.73 m^2^, prior renal transplant, obstructive nephropathy, acute glomerulonephritis, volume depletion, non-oliguria, active bleeding, pregnancy, and prior allergy to frusemide were excluded from the study.

### Study Design

Informed consent was obtained from each participant. The basic demographic data of the patients and their clinical history were recorded. The presence of diabetes, hypertension, or other co-morbidities and the treatment they pursue for these ailments were noted. Basic laboratory investigations were also done.

### Frusemide Stress Test

For frusemide-naïve patients, an intravenous bolus dose of 1 mg/kg of frusemide was administered and for patients who had received frusemide within the past week were given a bolus dose of 1.5 mg/kg. Urine output was then measured for the next two hours. Unless volume loss was considered desirable, urine output was replaced by the same volume with either normal saline or ringer's lactate solution for the first six hours. Patients' heart rate and blood pressure were monitored regularly during this time period and urine output monitoring was continued for 24 hours. Follow up was done for a period of 14 days post-FST or the patient was censored at death, whichever occurred first.

### Outcomes

**Figure 1:**
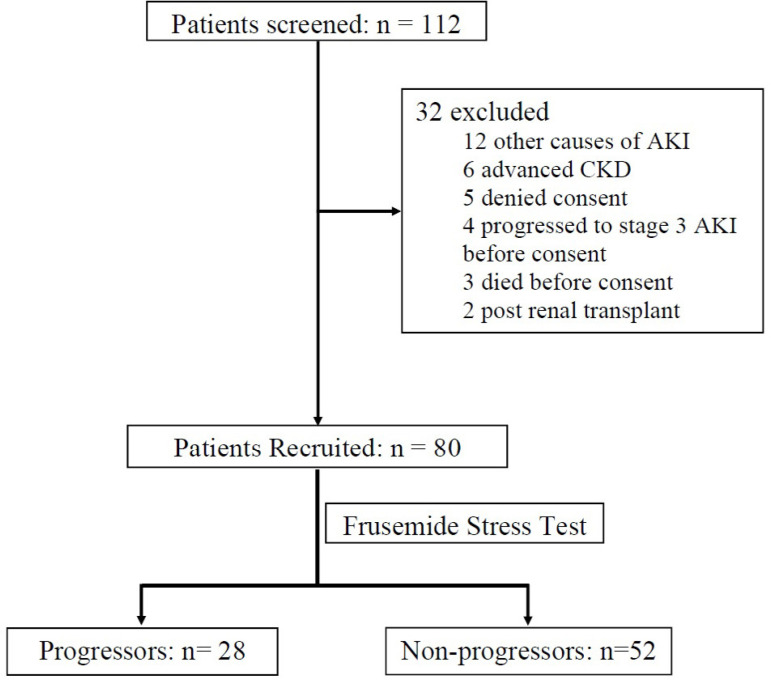
Study flow diagram.

The progression to AKI-KDIGO stage 3 (an increase in creatinine from baseline value by 3 times or serum creatinine ≥4 mg/dL or initiation of RRT or a urine output < 0.3 mL/kg/hr for ≥24 hours or anuria for ≥12 hours) within 14 days of FST was studied as the primary outcome. The patients were grouped into "progressors" and "non-progressors" depending on their progression to AKI-KDIGO stage 3. The composite end-point of achieving AKI-KDIGO stage 3 or death within 14 days of FST was studied as the secondary outcome. In addition, any adverse events related to frusemide were also recorded. The study flow diagram is shown in Figure 1.

### Statistics

To describe the data, descriptive statistics, frequency analysis, and percentage analysis were used for categorical variables and mean, standard deviation (SD) and standard error of mean (SE) were used for continuous variables. Differences between proportions of patients in each group with certain characteristics were assessed with the chi-square, Fisher exact, and Student t test as appropriate. The area under the curve (AUC) of the receiver operating characteristics (ROC) analysis was used to compare the 2-hour post-FST urine output response with the primary and secondary outcomes. Sensitivity, specificity, and optimal urine output cut-off using Youden index were also deduced from the curve. In all the above statistical tools, p ≤0.05 was considered significant. Statistical analysis was done using SPSS statistics software 23.0 version and MedCalc Statistical Software version 19.1.3.

## Results

### Baseline Characteristics

Among 112 patients screened for eligibility, 80 patients were recruited and underwent FST. The mean age of the participants was 52±1.67 years; 42 (52.5%) of the total cohort were male. Of the 80 patients, 28 (35%) met the primary outcome of AKI-KDIGO stage 3 and 34 (42.5%) met the secondary composite outcome of AKI-KDIGO stage 3 or death within 14 days of FST. The baseline characteristics of gender (*p*=0.425), diabetes mellitus (*p*=0.13), hypertension (*p*=0.104), cardiac failure (*p*=1.0), albumin (*p*=0.13), alcohol (*p*=0.587), and smoking status (*p*=0.977) were not significantly different between the patients who progressed and those who did not. There were six patients with baseline CKD in the entire cohort and when compared between the two groups, baseline CKD status was significantly associated with the progressors group (*p*=0.018). Regarding the etiology, 81.3% of patients of the entire cohort had sepsis as the predominant cause and no significant association (*p*=0.236) with either of the groups was found. The proportion of patients with AKI-KDIGO stage 1 and stage 2 was similar in both the groups (57.1 vs. 53.8% and 42.9 vs. 46.2%, *p*=0.78). The progressors group had a statistically higher mean pre-FST creatinine when compared to the non-progressors (*p*=0.05). Eight patients of the progressors needed RRT. During the study period, 16 (20%) of the 80 patients died. When compared between groups, the progressors group had 35.7% mortality while the non-progressors had 11.5% (*p*=0.01). The patient characteristics and outcomes are summarized in Table 1.

**Table 1:** Patient characteristics and outcomes

Characteristics	Combined (n=80)	Progressors (n=28)	Non-progressors (n=52)	p
**Age (years, mean ± SE)**	52±1.67	52.86±2.92	51.56±2.05	0.609
**Gender (male), n (%)**	42(52.5%)	13(46.4%)	29(55.8%)	0.425
**Comorbidities, n (%)**				
Diabetes mellitus	31(38.8%)	14(50%)	17(32.7%)	0.13
Hypertension	20(25%)	10(35.7%)	10(19.2%)	0.104
Cardiac failure	9(11.3%)	3(10.7%)	6(11.5%)	1.0
CKD	6(7.5%)	5(17.9%)	1(1.9%)	0.018
Albumin (g/dL, mean ± SD)	-	3.62±0.32	3.73±0.3	0.13
**Smoking, n (%)**	17(21.3%)	6(21.4%)	11(21.2%)	0.977
**Alcohol intake, n (%)**	23(28.8%)	7(25%)	16(30.8%)	0.587
**Sepsis, n (%)**	65(81.3%)	25(89.3%)	40(76.9%)	0.236
**Clinical data**				
Pre-FST creatinine (mg/dL)	-	2.07±0.09	1.86±0.05	0.05
Frusemide (1.5mg/kg), n (%)	15(18.8%)	8(28.6%)	7(13.5%)	0.099
2-hour post-FST urine output (mL, mean ± SE)	-	212.86±18.98	524.81±30.03	<0.001
AKI-KDIGO 1, n (%)	44(55%)	16(57.1%)	28(53.8%)	0.777
AKI-KDIGO 2, n (%)	36(45%)	12(42.9%)	24(46.2%)	
**Outcomes, n (%)**				
AKI-KDIGO 3	28(35%)	28(100%)	-	-
Death	16(20%)	10(35.7%)	6(11.5%)	0.01
RRT	8(10%)	8(28.6%)	-	-

Abbreviations: CKD: Chronic Kidney Disease, FST: Frusemide Stress Test, AKI-KDIGO: Acute Kidney Injury-Kidney Disease: Improving Global Outcomes, RRT: Renal Replacement Therapy.

### Frusemide Stress Test

**Figure 2:**
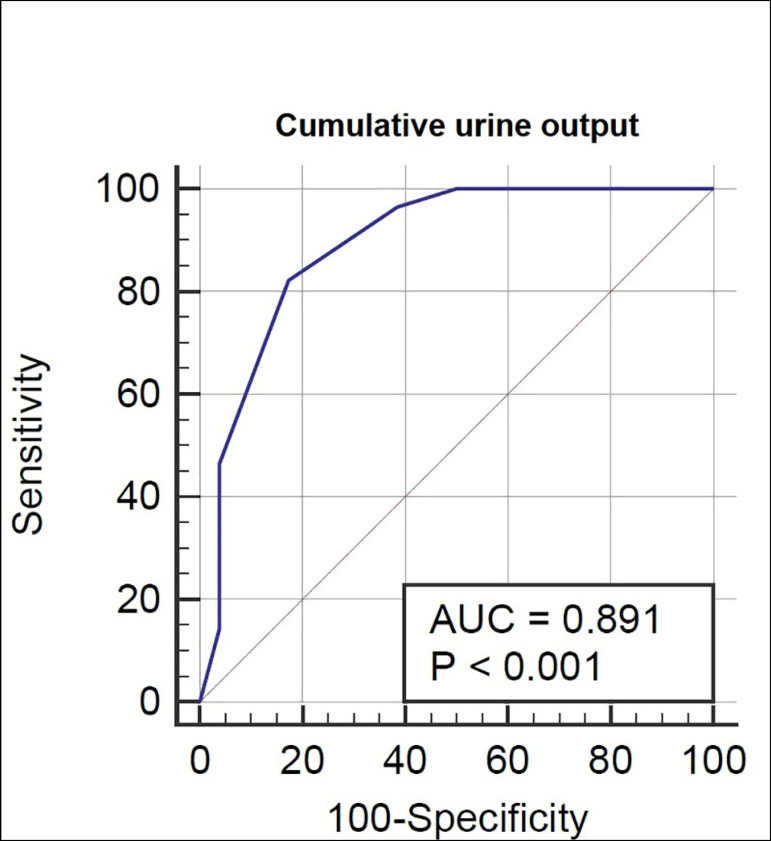
ROC curve of cumulative 2-hour post-FST urine output to predict the primary outcome of progression to AKI-KDIGO stage 3.

**Figure 3:**
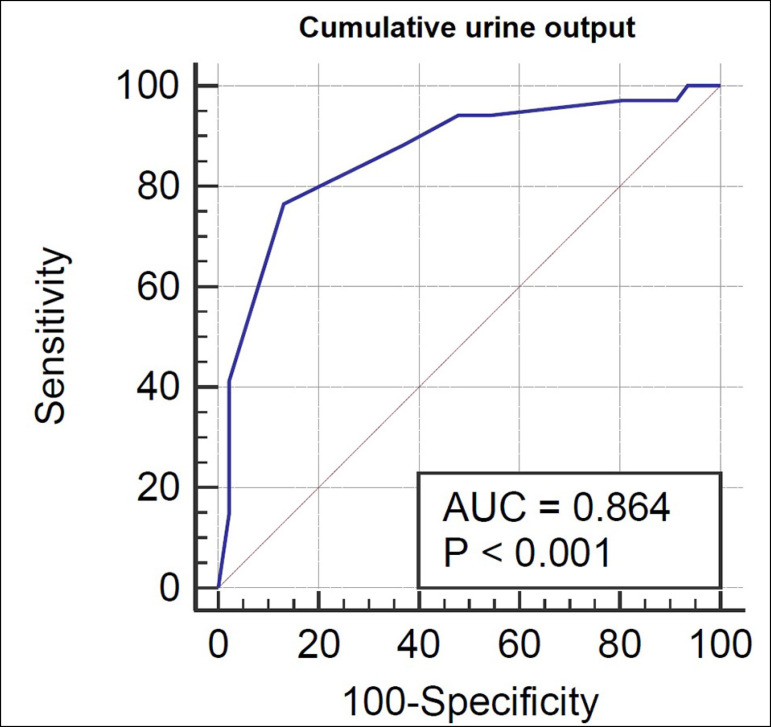
ROC curve of cumulative 2-hour post-FST urine output to predict the secondary composite outcome of AKI-KDIGO stage 3/ death.

The test procedure was well tolerated by the patients without any adverse events related to the test. There was no significant difference among patients who received an augmented frusemide dose (1.5 mg/kg) between the two groups (*p*=0.09). Between groups, patients who progressed to stage 3 had significantly lower cumulative 2-hour post-FST urine output (212.86±18.98 mL) compared to those who did not progress (524.81±30.03 mL) with a *p*<0.0001. On analysis of the ROC curve regarding the primary outcome, the cumulative 2-hour urine output after FST had an AUC of 0.89±0.03 (*p*<0.001) (Figure 2). The sensitivity and specificity of various 2-hour post-FST urine output cut-offs for predicting progression was also assessed (Table 2). A cumulative 2-hour post-FST urine output of ≤ 300 mL as determined by the Youden index had 82.14% sensitivity and 82.69% specificity for predicting progression (Table 4). As for the secondary composite outcome of AKI-KDIGO stage 3 or death, the cumulative 2-hour urine output after FST had an AUC of 0.86±0.04 (*p*<0.001) (Figure 3). Among the various urine volume cut-offs, a cumulative 2-hour post-FST urine output of ≤ 300 mL had 76.47% sensitivity and 86.96% specificity for predicting the secondary outcome (Table 3 and 4).

**Table 2:** Sensitivity and specificity of cumulative 2-hour post-FST urine output thresholds for progression to AKI-KDIGO stage 3

Cumulative 2-hour urine output	Sensitivity	Specificity
≤ 100 mL	14.29	96.15
≤ 200 mL	46.43	96.15
≤ 300 mL	82.14	82.69
≤ 400 mL	96.43	61.54
≤ 500 mL	100.00	50.00

FST: Frusemide Stress Test.

**Table 3:** Sensitivity and specificity of cumulative 2-hour post-FST urine output thresholds for progression to AKI-KDIGO stage 3/death

Cumulative 2-hour urine output	Sensitivity	Specificity
≤ 100 mL	14.71	97.83
≤ 200 mL	41.18	97.83
≤ 300 mL	76.47	86.96
≤ 400 mL	88.24	63.04
≤ 500 mL	94.12	52.17

FST: Frusemide Stress Test.

**Table 4:** Optimal urine output cut-off characteristics for primary and secondary outcomes

Outcome	Urine output cut-off	Sensitivity	Specificity	Youden index J	Area Under Curve (standard error)	p value
Primary outcome	≤ 300 mL	82.14	82.69	0.6343	0.86(0.04)	<0.001
Secondary outcome	≤ 300 mL	76.47	86.96	0.6484	0.89(0.03)	<0.001

## Discussion

AKI does not manifest specific signs and symptoms in the immediate aftermath of an inciting insult, and when signs of reduced kidney function manifest, substantial damage has already occurred mitigating the time window for intervention^3^. Therefore, an unmet need exists for AKI prediction early enough to improve outcomes. Since most forms of AKI involve acute tubular injury, it has been surmised that biomarkers of tubular integrity will better predict risk of AKI progression^5^. The first studies utilized measurement of tubular creatinine secretion as surrogate for tubular functional assessment^4^. Because of unstable creatinine kinetics in AKI, apart from its inherent flaws, its utility in assessing tubular functional capacity in AKI becomes questionable^4^. The loop diuretic frusemide shows promise since multiple domains of the nephron tubule must be functionally intact for frusemide induced increase in urinary output to occur. Frusemide, being an organic anion, circulates bound to albumin and hence not freely filtered across the glomerular barrier. The drug gains access to the tubular lumen by secretion via human organic anion transporter in the proximal convoluted tubule, acts in the thick ascending loop of Henle by inhibiting luminal Na^+^K^+^2Cl^-^ cotransporter, and the distal tubular lumen patency is required for urinary flow^7^^-^ 9. Apart from reducing fluid overload, diuretic use in AKI has not been shown to improve patient-centered outcomes in terms of prevention, RRT requirement, or recovery, though few animal studies have shown beneficial effects of frusemide by means of decreasing medullary oxygen consumption and modulating tubular cell survival^1^^,^ 10 ^-^ 13.

The idea of using frusemide to test tubular integrity is not an entirely new concept. Baek *et al*. in 1973 found that poor diuretic response and near zero free water clearance after frusemide challenge in patients without clinically apparent AKI signaled imminent AKI^14^.

In our study, we found that a cumulative 2-hour post-FST urine output of 300 mL or less predicted progression to AKI-KDIGO stage 3 (AUC 0.89) with a sensitivity of 82.14% and specificity of 82.69%. For the composite outcome of AKI-KDIGO stage 3 or death, cumulative 2-hour post-FST urine output of ≤300 mL had 76.47% sensitivity, 86.96% specificity, and AUC of 0.86. We did not encounter any adverse event related to the test.

In their seminal paper, Chawla *et al.* demonstrated that a cumulative 2-hour urine output following FST had the best predictive ability (AUC 0.87), and a urine output of ≤200 mL at 2 hours reliably predicted progression to severe AKI with 87.1% sensitivity and 84.1% specificity in 77 patients with early AKI (stage 1 and stage 2) assembled from two cohorts^5^. The cumulative 200 mL urine output criterion was prospectively validated in a multicentric study of 92 critically ill patients by Rewa *et al.* with sensitivity of 73.9%, specificity of 90.0%, and AUC of 0.87.^15^ The reason for the disparity in optimal urine output criteria between our study and the other studies mentioned could be that the aforementioned studies had higher number of patients with baseline CKD (n=24 in Chawla *et al.* and n=21 in Rewa *et al*.) compared to ours (n=6) and the frusemide response might have been poor in pre-existing tubular damage. Also, the number of patients with AKI stage 2 was higher in the progressors group in the study by Chawla *et al*.. Elsaegh *et al*. also found 2-hour urine output post-FST ≤200 mL to best predict AKI progression in sepsis patients with 89.29% sensitivity and 93.75% specificity. However, data on baseline CKD status was not provided^16^.

The performance of the FST has also been compared with existing structural damage biomarkers in few studies. In the same cohort of 77 patients studied by Chawla *et al.,* Koyner *et al.* compared the predictive ability of FST urine output with a panel of biomarkers to predict stage 3 AKI, receipt of RRT, and inpatient mortality. For each of the end-points studied, FST outperformed all biomarkers for prediction, and combining FST with individual biomarkers in logistic regression did not significantly improve risk prediction. However, the study revealed an interesting inference that, in patients with elevated biomarkers, the AUC for FST urine output to predict progression to stage 3 increased from 0.87 to 0.90 and that for RRT, AUC increased from 0.86 to 0.91. This study showed that in high-risk patients, FST further improves risk stratification.^6^ Matsuura *et al.* demonstrated in a retrospective study of 95 ICU patients that response to variable dose of frusemide (frusemide responsiveness, FR) predicts progression to stage 3 AKI better than plasma NGAL (AUC 0.87 vs. 0.80). For predicting severe AKI, FR of 3.9 mL urine output for each mg of frusemide in two hours had the best discriminating capacity. Additionally, in patients with elevated plasma NGAL levels, FR showed favorable efficacy to predict progression (AUC-0.84)^17^.

Apart from recognizing early AKI, the ideal timing of RRT is intensely debated, with pros and cons for early as well as late initiation, and even large-scale studies concerning this issue have produced contrasting results.^1^ Lumlertgul *et al.* utilized FST as a screening tool to predict the need for RRT in a cohort of 162 patients. Of the 44 patients with positive FST response, only six (13.6%) needed RRT. The remaining 118 FST non-responsive patients were randomized to early versus standard (indication driven) RRT initiation. Forty five out of the 60 patients (75%) in the standard arm received RRT^18^.

Apart from prospective risk stratification, response to frusemide has also been shown to predict AKI recovery. Van der voot *et al.* utilized a version of the FST and showed that diuretic response to FST predicted renal recovery in patients undergoing continuous renal replacement therapy (AUC=0.84)^19^.

The FST has proved to be an excellent functional biomarker for AKI risk stratification. However, if the biomarker is applied to a broader population, the number of false positives will be high, and the biomarker performance will decrease^2^. For enhanced biomarker performance, the population at risk of renal injury needs to be defined. Based on these facts, a new algorithm for AKI approach can be formulated to improve patient outcomes. First, patients at risk for AKI need to be identified such as elderly, presence of diabetes mellitus, chronic kidney disease, organ failure, etc. and monitored for 'renal angina', i.e., early signs of injury such as subtle increase in creatinine (e.g., 0.1 mg/dL creatinine rise as opposed to 0.3 mg/dL, which will qualify as AKI), fluid overload, and reduction in urine output^2^^,^ 3. Combing the risk factors and renal angina manifestations, the 'renal angina index' for use in pediatric and adult population with excellent negative predictive value have been formulated.^3^ This assessment should be followed by testing for structural damage biomarker and in patients in whom the biomarker tests positive, FST can be used to further improve risk stratification.^3^ This model of serial testing with initial tests having good negative predictive value followed by biomarkers with higher positive predictive value will provide better risk stratification of early AKI patients^2^^,^ 3.

### Clinical Implications

In a real-world setting, the application of FST can be viewed in two spectrums. In resource-limited places, early AKI patients who fail FST need to be flagged for vigilant monitoring for identifying progression early to improve outcomes. The 18th acute dialysis quality initiative (ADQI) consensus Conference focusing on "Management of AKI in the Developing World", have given a "we recommend" status to perform FST after adequate fluid resuscitation under monitored conditions (not graded)^20^. They have also advised to develop a concise version of the FST with easy applicability. In resource rich and research settings, FST can be used as a tool to identify patients at risk of progression and these patients can be subjected to measurement of newer biomarkers or be enrolled in trials for testing novel intervention strategies to address the basic pathophysiology^2^.

Apart from prospective AKI risk stratification, FST is also being assessed in other domains. McMahon *et al.* and Udomkarnjananun *et al.* have utilized different versions of FST to predict delayed graft function in renal transplant recipients in the immediate post-op period^21^^,^ 22. Kalra *et al.* used FST to assess intravascular volume status in nephrotic children to decide whether diuretic alone or albumin with diuretic is needed for edema management and to rationalize diuretic use^23^.

### Limitations

This was a pilot study with a small sample size. The urine output cut-off needs to be validated in multicenter, prospective studies with a large cohort. Though numerically small, baseline CKD status was statistically associated with the progressors group, which might have affected the study outcome. We also did not compare the performance of FST with other biomarkers due to logistic reasons. Also, long term follow-up of the patients after discharge was not done. Nevertheless, our study is one of the first studies conducted in our country to study the utility of FST.

## Conclusion

We conclude that FST as a tubular integrity biomarker shows excellent predictive capacity for prospective risk stratification of early AKI. As a novel dynamic biomarker on the horizon, FST needs to be incorporated as part of decision-making tools for identifying AKI early enough to try novel interventions to mitigate the negative ramifications of this global health problem.
